# Supplementary material for: Bis-Schiff base linkage-triggered highly bright luminescence of gold nanoclusters in aqueous solution at the single-cluster level
Source: Nat Commun. 2022 Jun 13;13:3381. doi: 10.1038/s41467-022-30760-3 (PMC9192726; doi:10.1038/s41467-022-30760-3)
Supplement: Supplementary file 1 — Supplmentary Information [file 41467_2022_30760_MOESM1_ESM.pdf]

Supplementary Information

Bis-Schiff base linkage-triggered highly bright luminescence of gold nanoclusters in aqueous solution at the single-cluster level

Haohua Deng et al.

Supplementary Method

Materials. $HAuCl_4 \cdot 3H_2O$ (≥99.9%), $NaBH_4$ (98%), glutaraldehyde (GA, 50 wt.% aqueous solution), furan-2,5-dicarbaldehyde (DFF, >98.0%), m-phthalaldehyde (mPA, 98.0%), 2,6-pyridinedicarboxaldehyde (PDA, >97.0%) and ammonium persulphate (APS, ≥98.0%) were obtained from Aladdin Reagent Company (Shanghai, China). L-Glutathione (SG, ≥98.0%) was purchased from Sigma-Aldrich Reagent Co. (Shanghai, China). An aqueous solution of 40% (w/v) acrylamide/bis-acrylamide (19:1) was brought from Beijing Dingguo Changsheng Biotechnology Co., Ltd (Beijing, China). N,N,N',N'-Tetramethylethylenediamine (TEMED, 99%) was obtained from Beyotime Institute of Biotechnology (Shanghai, China). The reagents and chemicals were used as received without further purification. Ultrapure water was used throughout the work.

Instruments. UV-visible (UV-vis) absorption spectra were conducted on a Shimadzu UV-2450 spectrophotometer (Shimadzu, Japan). Photoluminescence spectra were recorded using a Cary Eclipse spectrophotometer (Agilent, USA). Luminescence lifetime experiments were carried out by time-correlated single-photon counting (TCSPC) on a F900 luminescence spectrometer (Edinburgh, UK). Native PAGE was performed on a Bio-Rad Mini-PROTEAN Tetra system. The isolated $Au_{22}(SG)_{18}$ NCs were analyzed by using electrospray ionization time-of-flight mass spectrometry (ESI-TOF-MS) (Q Exactive; Thermo Fisher Scientific, USA). The transmission electron microscopy (TEM) images were recorded in a JEM-2100 electron microscope (JEOL, Japan). Dynamic light scattering (DLS) measurements were performed on a Zetasizer Nano ZS (Malvern Instruments, UK). Fourier transform infrared (FT-IR) spectra were recorded on a Nicolet Avatar 360 FT-IR spectrophotometer (Thermo Fisher Scientific, USA). ^{1}H nuclear magnetic resonance (^{1}H NMR) spectra were obtained using a Bruker 600 MHz AVANCE III NMR spectrometer (Bruker BioSpin, Germany). Electron spin resonance (ESR) analysis was conducted on a JEOL JES-FA200 ESR spectrometer (ESP-300E, Bruker, Germany).

Transient absorption (TA) measurements. TA spectra were measured by optical pump-probe spectroscopy. The output of a mode-locked Ti-sapphire laser amplifier (Spectra-Physics) was used as a source of femtosecond radiation (800 nm, 35-40 fs, 1 kHz, an average power of 4 W). A home-built pump-probe setup was used for obtaining transient absorption spectra and kinetics. The required pump pulse was generated by using an Optical Parametric Amplifier (TOPAS, Light conversion). White light continuum (350-850 nm) generated in 3 mm thickness rotated CaF_2 plate was used as probe beam. The experimental data were fitted to a multi-exponential decay function convoluted with the instrument response function. The overall time resolution was about 20-30 fs.

Pump-probe measurements were performed in 1 mm path length quartz cuvettes under continuous movement for homogeneous irradiation during exposure. The power was controlled 0.3 mW at 400 nm excitation.

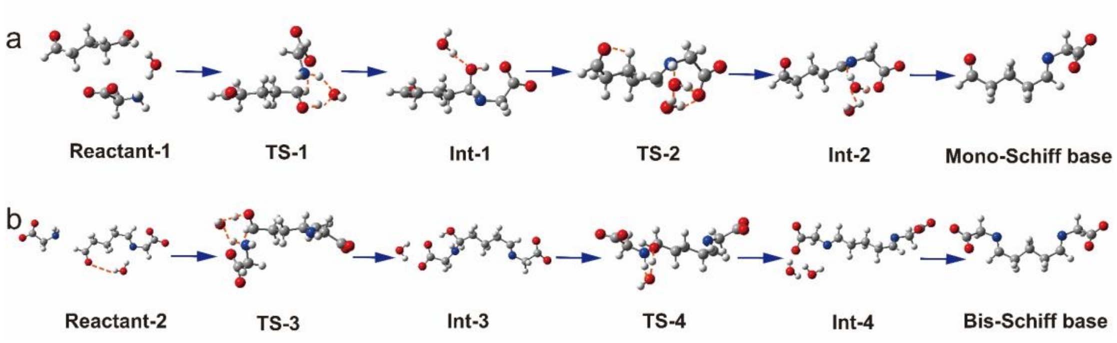

Supplementary Fig. 1 Theoretical investigations of the bis-Schiff base formation reaction for GA. (a) The formation processes of mono-Schiff of GA. (b) The formation processes of bis-Schiff bases of GA. TS: transition state, Int: intermediate. The gray, white, red, and blue balls represent C, H, O, and N atoms, respectively.

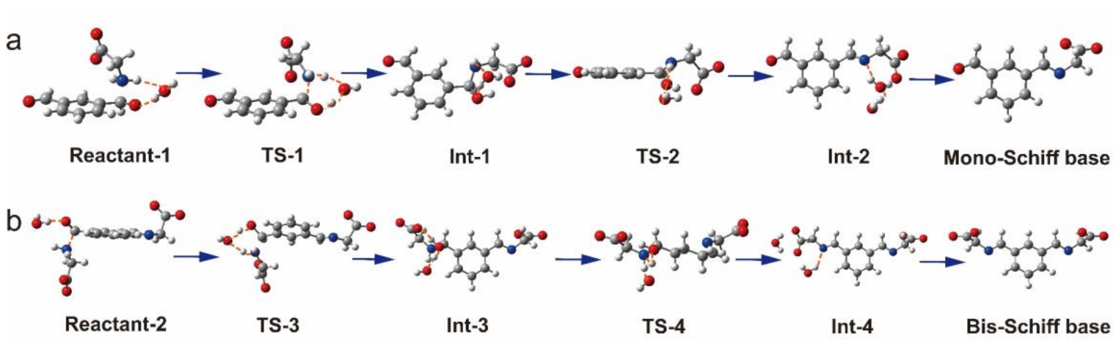

Supplementary Fig. 2 Theoretical investigations of the bis-Schiff base formation reaction for mPA. (a) The formation processes of mono-Schiff of mPA. (b) the formation processes of bis-Schiff bases of mPA.

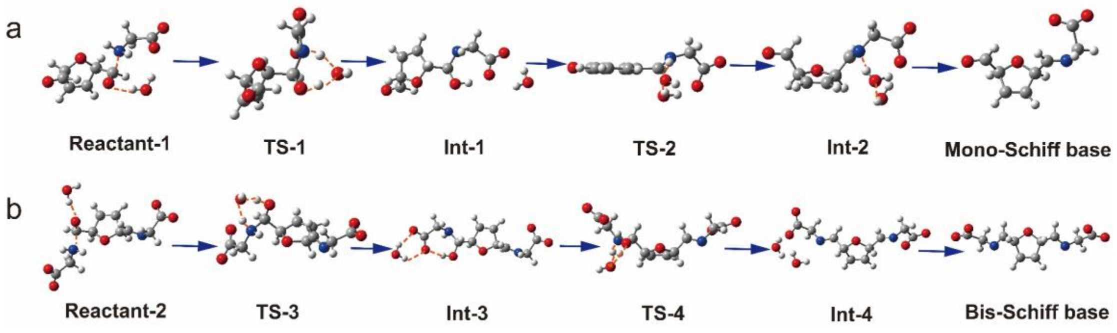

Supplementary Fig. 3 Theoretical investigations of the bis-Schiff base formation reaction for DFF. (a) The formation processes of mono-Schiff of DFF. (b) The formation processes of bis-Schiff bases of DFF.

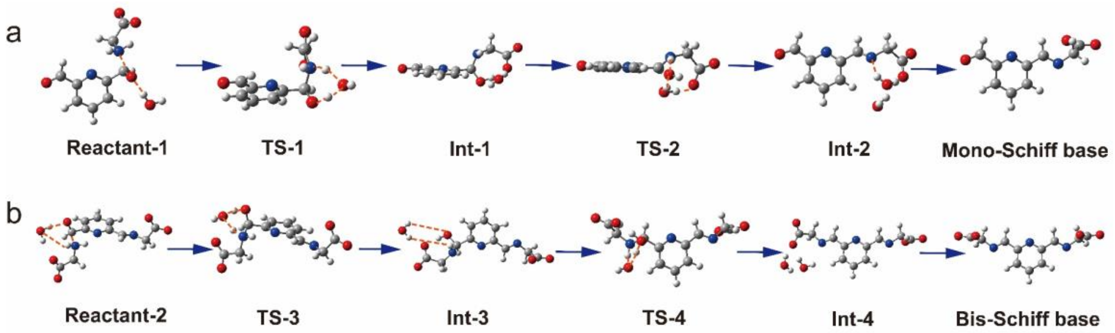

Supplementary Fig. 4 Theoretical investigations of the bis-Schiff base formation reaction for PDA. (a) The formation processes of mono-Schiff of PDA. (b) the formation processes of bis-Schiff bases of PDA.

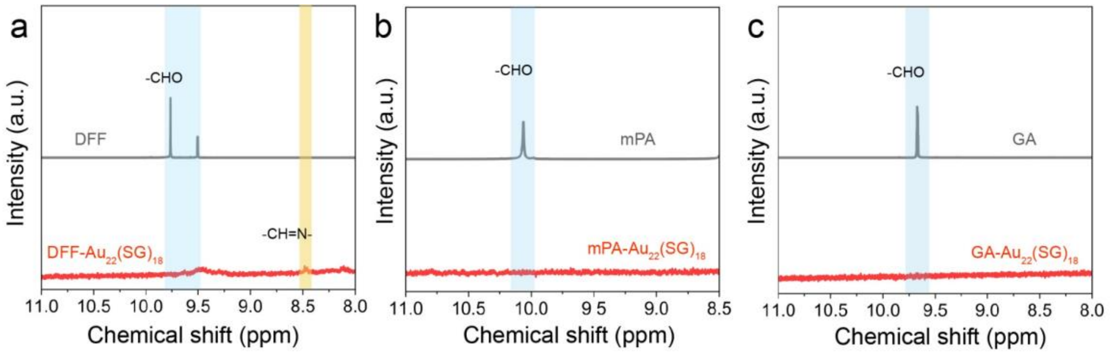

Supplementary Fig. 5 ^{1}H NMR measurements. (a) ^{1}H NMR spectra of DFF and DFF-$Au_{22}(SG)_{18}$ NCs. (b) ^{1}H NMR spectra of mPA and mPA-$Au_{22}(SG)_{18}$ NCs. (c) ^{1}H NMR spectra of GA and GA-$Au_{22}(SG)_{18}$ NCs. a.u., arbitrary units.

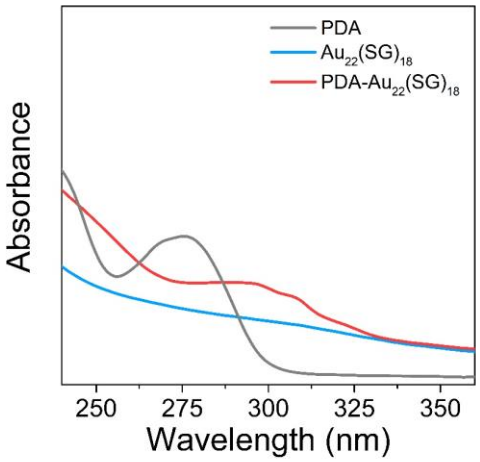

Supplementary Fig. 6 UV absorption spectra of PDA, $Au_{22}(SG)_{18}$ NCs, and PDA-$Au_{22}(SG)_{18}$ NCs.

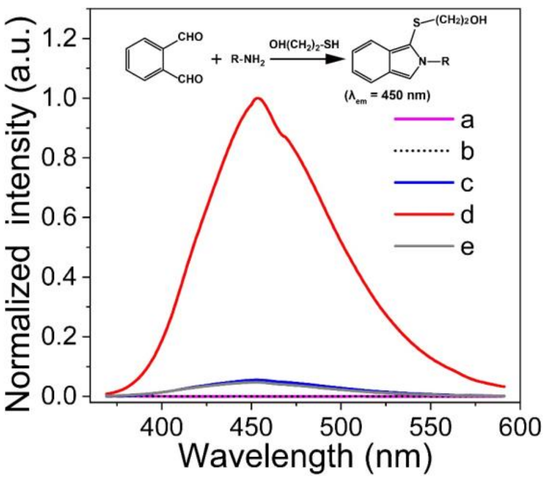

Supplementary Fig. 7 Emission spectra of (a) $Au_{22}(SG)_{18}$, (b) PDA-$Au_{22}(SG)_{18}$, (c) *o*-phthalaldehyde (OPA) + 2-mercaptoethanol (2-ME), (d) $Au_{22}(SG)_{18}$ + OPA + 2-ME, and (e) PDA-$Au_{22}(SG)_{18}$ + OPA + 2-ME. Inset shows the reaction between SG molecule and amino-reactive reagent (OPA and 2-ME). The reactions were conducted at 37 °C for 10 min, and the excitation wavelength was chosen as 340 nm. a.u., arbitrary units.

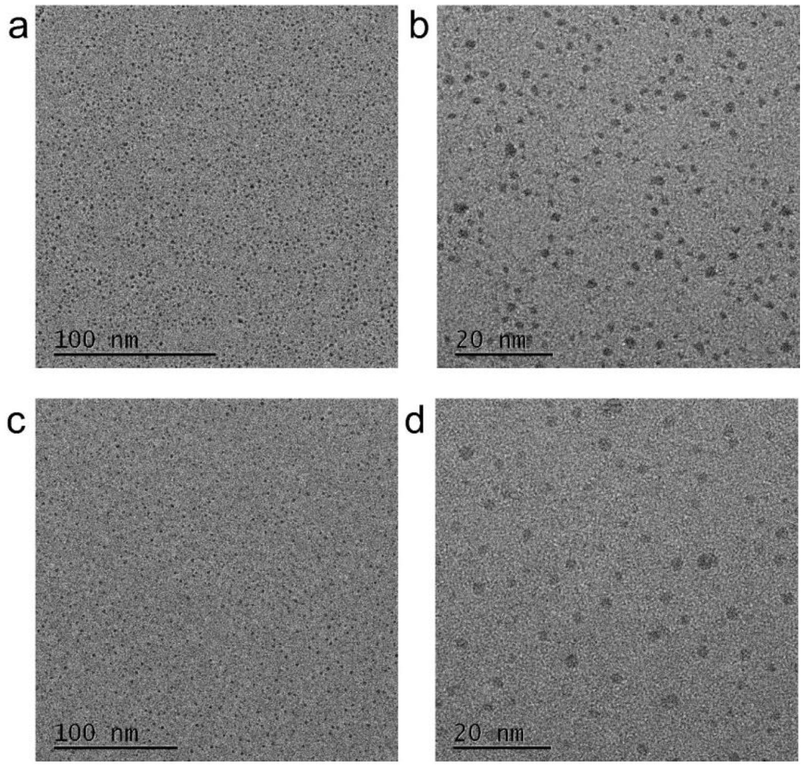

Supplementary Fig. 8 TEM images of $Au_{22}(SG)_{18}$ (a and b) and PDA-$Au_{22}(SG)_{18}$ (c and d) NCs.

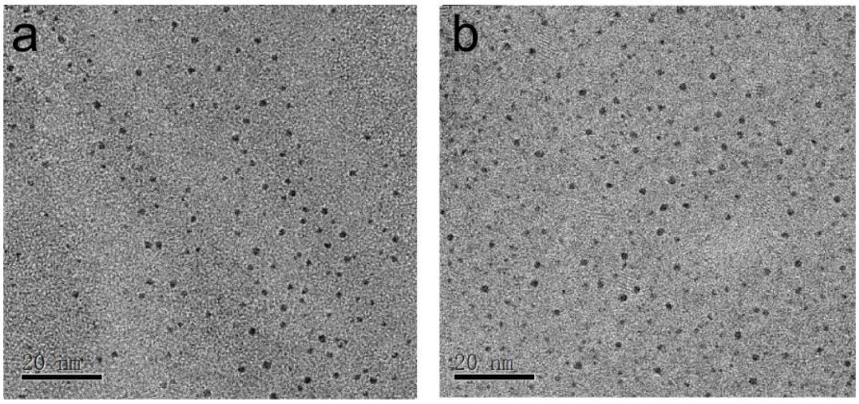

Supplementary Fig. 9 TEM images of (a) mPA-$Au_{22}(SG)_{18}$ and (b) DFF-$Au_{22}(SG)_{18}$ NCs.

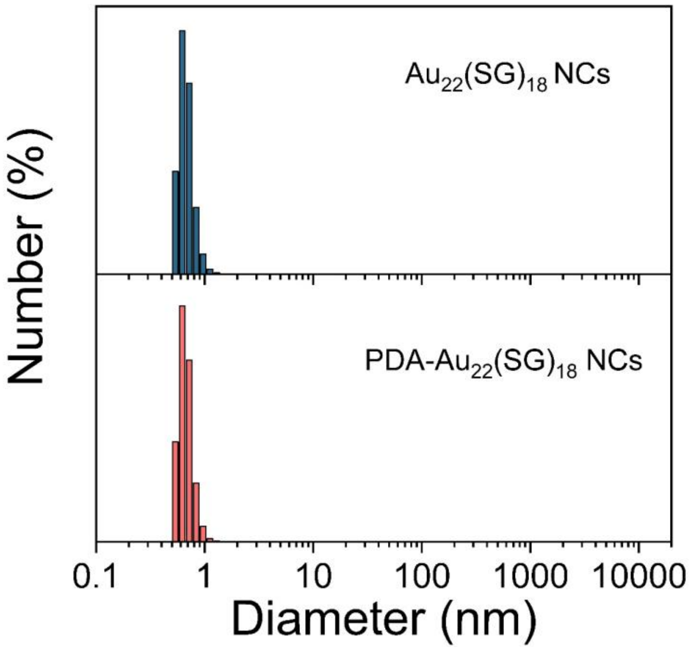

Supplementary Fig. 10 DLS measurements of $Au_{22}(SG)_{18}$ and PDA-$Au_{22}(SG)_{18}$ NCs.

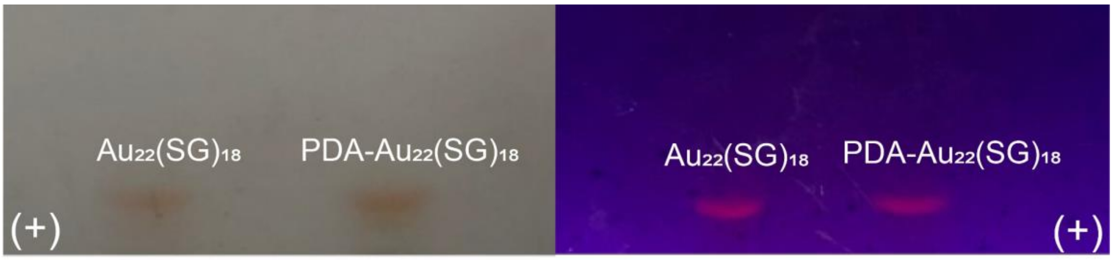

Supplementary Fig. 11 Digital photos of the PAGE gels of $Au_{22}(SG)_{18}$ and PDA-$Au_{22}(SG)_{18}$ NCs under visible (left) and UV (right) lights.

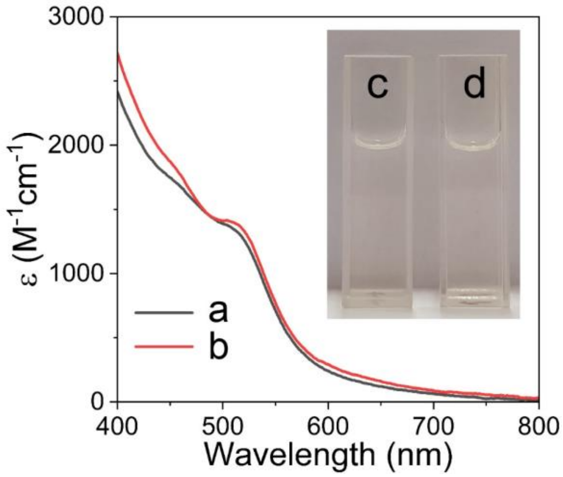

Supplementary Fig. 12 Absorption spectra of (a) $Au_{22}(SG)_{18}$ and (b) PDA-$Au_{22}(SG)_{18}$ NCs. The inset: photographic images of (c) $Au_{22}(SG)_{18}$ and (d) PDA-$Au_{22}(SG)_{18}$ NCs.

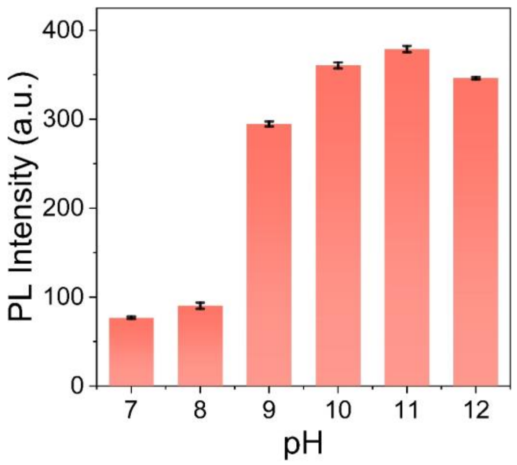

Supplementary Fig. 13 The pH-dependent photoluminescence intensity of PDA-$Au_{22}(SG)_{18}$ NCs. Error bars represent standard deviation over three independent measurements. a.u., arbitrary units.

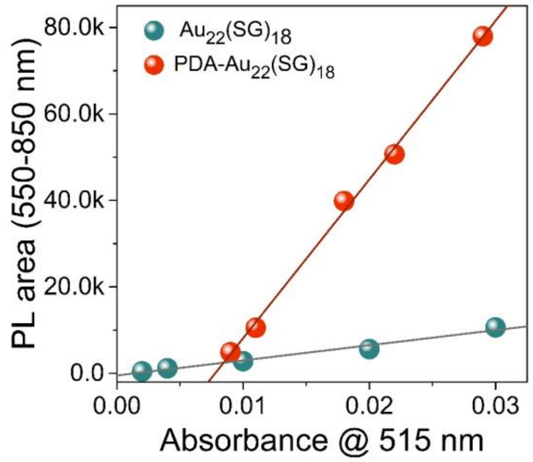

Supplementary Fig. 14 Absorbance-dependent emission area plots for $Au_{22}(SG)_{18}$ and PDA-$Au_{22}(SG)_{18}$ NCs. The PL quantum yield (QY) of PDA-$Au_{22}(SG)_{18}$ was calculated as follows:

$$QY = QY_{Ref} \frac{Grad}{Grad_{Ref}} \frac{n^2}{n_{Ref}^2}$$

where QY_{Ref} is the luminescence QY of the reference $Au_{22}(SG)_{18}$ NCs (absolute QY = 4.6%), Grad is the gradient from the plot of integrated emission intensity (550-850 nm) vs absorbance (515 nm) for PDA-$Au_{22}(SG)_{18}$ NCs, $Grad_{Ref}$ is the gradient from the plot of integrated emission intensity (550-850 nm) vs absorbance (515 nm) for $Au_{22}(SG)_{18}$ NCs, n is the refractive index of the solvent for PDA-$Au_{22}(SG)_{18}$ NCs (H_2O, 1.333), and n_{Ref} is the refractive index of the solvent for $Au_{22}(SG)_{18}$ NCs (H_2O, 1.333).

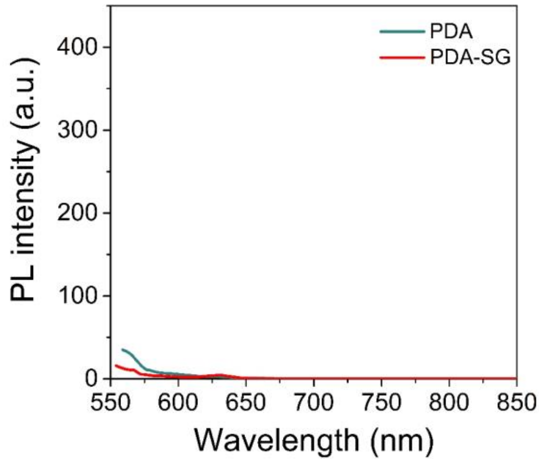

Supplementary Fig. 15 PL emission spectra of PDA and PDA-SG complex. a.u., arbitrary units.

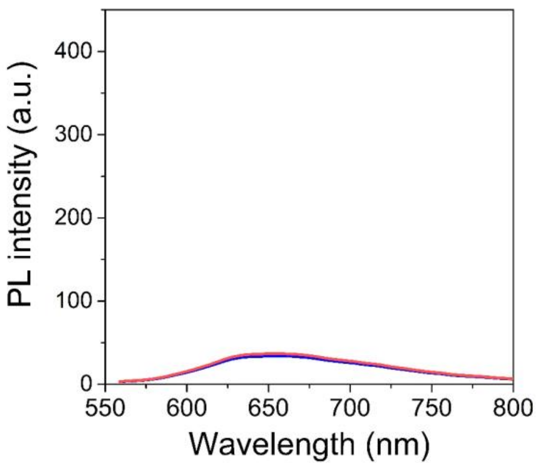

Supplementary Fig. 16 PL emission spectra of $Au_{22}(SG)_{18}$ in the absence (blue line) and presence (red line) of 2-pyridinecarboxaldehyde. a.u., arbitrary units.

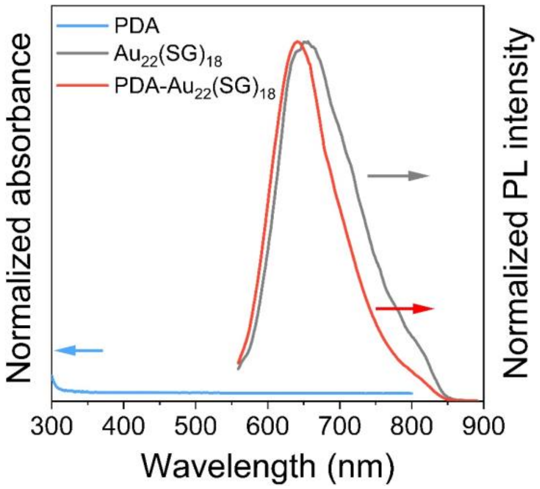

Supplementary Fig. 17 Absorption spectrum of PDA (blue line), and emission spectra of $Au_{22}(SG)_{18}$ (gray line) and PDA-$Au_{22}(SG)_{18}$ (red line) NCs.

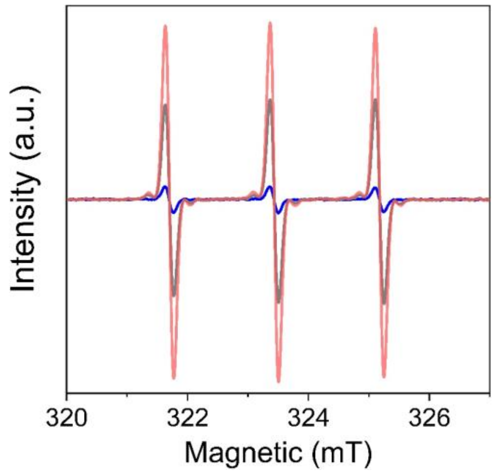

Supplementary Fig. 18 Electron spin resonance spectra of 2,2,6,6-tetramethylpiperidine (TEMP) (blue line), $Au_{22}(SG)_{18}$ NCs + TEMP (gray line), and PDA-$Au_{22}(SG)_{18}$ NCs + TEMP (red line) after 520 nm light illumination for 30 min. a.u., arbitrary units.

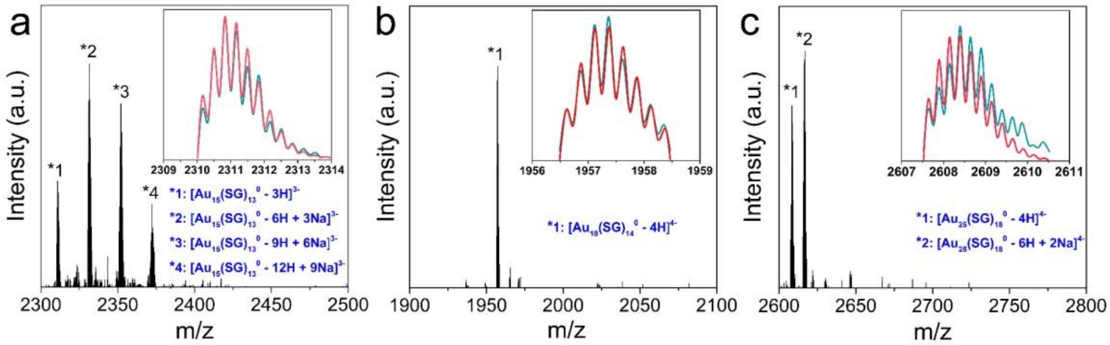

Supplementary Fig. 19 ESI mass spectra of other gold NCs. (a) ESI mass spectrum of $Au_{15}(SG)_{13}$ NCs. Inset shows the experimental (blue line) and simulated (red line) isotope patterns of $[Au_{15}(SG)_{13}^{0} - 3H]^{3-}$. (b) ESI mass spectrum of $Au_{18}(SG)_{14}$ NCs. Inset shows the experimental (blue line) and simulated (red line) isotope patterns of $[Au_{18}(SG)_{14}^{0} - 4H]^{4-}$. (c) ESI mass spectrum of $Au_{25}(SG)_{18}$ NCs. Inset shows the experimental (blue line) and simulated (red line) isotope patterns of $[Au_{25}(SG)_{18}^{0} - 4H]^{4-}$. a.u., arbitrary units.

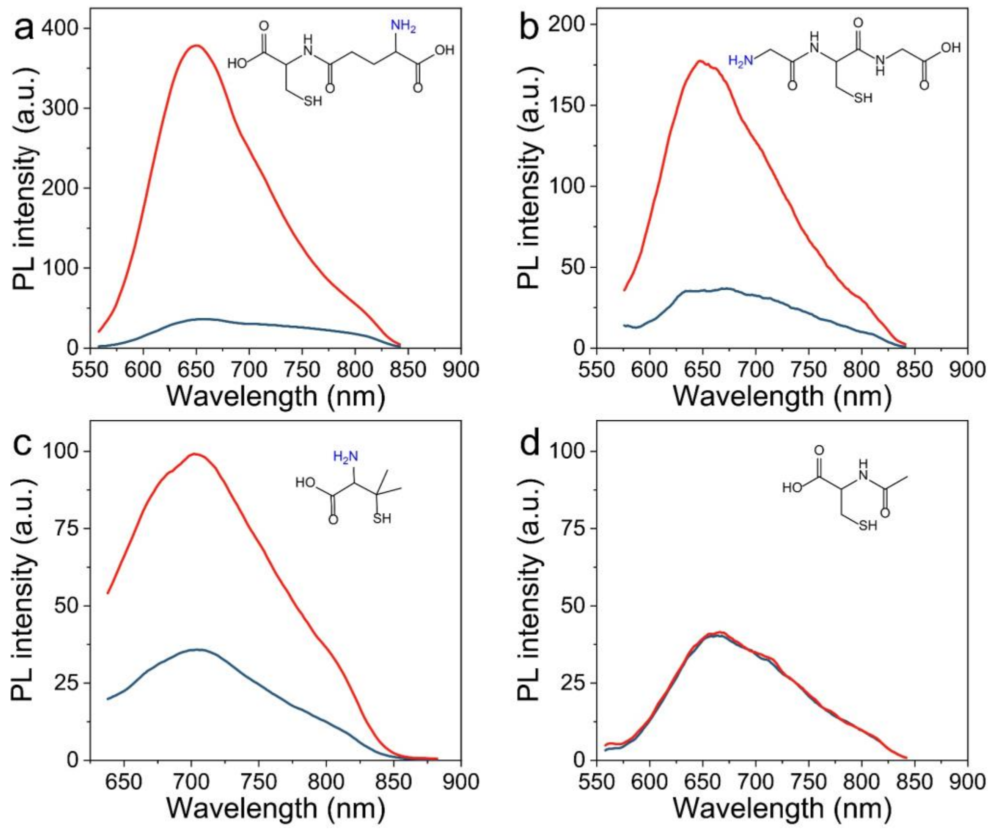

Supplementary Fig. 20 Generality of the proposed strategy for other ligand systems. The effect of PDA on the luminescence of gold NCs protected by (a) γ-Glu-Cys, (b) Gly-Cys-Gly, (c) L-penicillamine, and (d) N-acetyl-L-cysteine (blue line: gold NCs; red line: gold NCs + PDA). Inset shows the chemical structure of the corresponding ligand. a.u., arbitrary units.

Supplementary Table 1 Parameters obtained from PL measurements.

Sample	QY (%)	τ_1 (μs)	τ_2 (μs)	χ^2	τ_{ave} (μs)	k_R (s^{-1})	k_{nR} (s^{-1})
$Au_{22}(SG)_{18}$	4.6	0.9<br>(41.4%)	8.6<br>(58.6%)	1.27	5.4	8.5×10^3	1.8×10^5
PDA-$Au_{22}(SG)_{18}$	48±1.4	3.6<br>(50.3%)	15.1<br>(49.7%)	1.04	9.3	5.2×10^4	5.6×10^4

QY: quantum yield, τ_{ave}: average lifetime, k_R: radiative decay rate, k_{nR}: non-radiative decay rate.

The values in % indicate the relative amplitudes (A_i) of lifetimes.
