# Supplementary material for: Bis-Schiff base linkage-triggered highly bright luminescence of gold nanoclusters in aqueous solution at the single-cluster level
Source: Nat Commun. 2022 Jun 13;13:3381. doi: 10.1038/s41467-022-30760-3 (PMC9192726; doi:10.1038/s41467-022-30760-3)
Supplement: Supplementary file 3 — Description of Additional Supplementary Files [file 41467_2022_30760_MOESM3_ESM.docx]

**Description of Additional Supplementary Files**

File Name: Supplementary Data 1

Description: Cartesian coordinates for the calculated structures
