# Supplementary material for: Bis-Schiff base linkage-triggered highly bright luminescence of gold nanoclusters in aqueous solution at the single-cluster level
Source: Nat Commun. 2022 Jun 13;13:3381. doi: 10.1038/s41467-022-30760-3 (PMC9192726; doi:10.1038/s41467-022-30760-3)
Supplement: Supplementary file 4 — Supplementary Data 1 [file 41467_2022_30760_MOESM4_ESM.pdf]

Cartesian coordinates for the calculated structures

1. Cartesian coordinates for GA

(1) TS1

C	-0.58612300	0.03017800	1.07467500
C	-1.81705900	0.15173700	0.16902800
H	-0.24576800	-1.00833900	1.14074700
H	-0.85603800	0.33341900	2.09274500
H	-2.08638500	1.20528700	0.06634200
H	-1.59604800	-0.21217300	-0.84065500
C	-3.00156400	-0.63954400	0.71908400
H	-2.73991900	-1.69775100	0.87554200
H	-3.28757800	-0.28141000	1.71973200
C	-4.25558600	-0.63467300	-0.11227700
C	0.59240400	0.92143500	0.68878600
O	-4.40018700	-0.09134900	-1.18107300
O	0.23395500	2.24081100	0.58228900
H	-5.10395900	-1.20198400	0.33814100
H	1.43398100	0.72962500	1.35752800
C	1.52277100	-0.89284400	-1.03695800
H	0.59218400	-1.44825400	-1.16872800
H	2.03646700	-0.87523800	-2.00108900
H	0.47461700	0.86349700	-1.37045000
H	1.12060200	2.73032600	0.13371300
N	1.17276500	0.52747600	-0.70473500
C	2.42498600	-1.68067300	-0.01817300
O	2.58891300	-2.86500100	-0.37475400
O	2.84124300	-1.05471100	0.97501900
O	2.29123200	2.86828200	-0.54517600
H	1.95555900	1.24219100	-0.81802900

H	2.37271600	3.61051900	-1.14782200

(2) TS2

C	0.94214500	0.66891800	-1.11685900
C	2.01280100	-0.04595200	-0.28226400
H	1.09064000	0.41706200	-2.17974800
H	1.04774200	1.75369000	-1.04201200
H	1.75690400	0.00771000	0.78056900
H	2.04308000	-1.10749300	-0.54307700
C	3.38515400	0.58093800	-0.52500900
H	3.57804900	0.73616600	-1.60141600
H	3.44393500	1.58800500	-0.08934300
C	4.58055500	-0.18910400	-0.02931100
C	-0.50432900	0.34269300	-0.83101900
O	4.59038400	-1.33674800	0.34420600
O	-0.81593700	1.79718500	0.53861400
H	5.53462600	0.39037600	-0.05315600
H	-1.21461200	0.74839600	-1.54596300
C	-2.24744300	-1.27590300	-0.26225600
H	-2.37704600	-1.82317800	-1.20036600
H	-2.34351700	-2.00823700	0.54082000
H	-0.38937200	-0.84381000	0.74122700
H	-1.79436400	1.62567400	0.49495200
N	-0.87046000	-0.75893900	-0.20354000
C	-3.48176100	-0.30310600	-0.14375800
O	-4.56490500	-0.89590400	-0.27165400
O	-3.26963400	0.92499900	0.04524300
O	0.10113600	0.02303400	2.09108000
H	-0.26487600	-0.13334000	2.96527500
H	-0.46906200	1.19012100	1.31997000

(3) TS3

C	-1.04252500	-0.26927300	1.37349700
C	0.30145700	-0.46321000	0.65476300
H	-1.23174200	0.79345100	1.56674300
H	-0.99838900	-0.75420000	2.35596900
H	0.39230300	-1.50498800	0.33889600
H	0.35470700	0.14251900	-0.25675600
C	1.49072300	-0.09847600	1.54818000
H	1.41161300	0.94856000	1.87907000
H	1.44220800	-0.69106600	2.47435200
C	2.86887000	-0.28463800	0.95753000
C	-2.26388400	-0.89096300	0.68792700
O	-2.10948200	-2.23554100	0.48105900
H	3.69639700	-0.18229600	1.67259500
H	-3.18142900	-0.62107400	1.22032700
C	-2.77079500	1.21136100	-0.86007700
H	-1.91643600	1.75671800	-0.45605700
H	-2.81853200	1.42301400	-1.93084300
H	-1.66338900	-0.49477000	-1.25375400
H	-2.87320600	-2.53711300	-0.28338600
N	-2.50064800	-0.25645900	-0.71924200
C	-4.08350300	1.76208100	-0.19788300
O	-4.25338700	2.97608100	-0.44404900
O	-4.76701600	0.96096300	0.46805400
O	-3.74516000	-2.54019800	-1.29516800
H	-3.24979400	-0.88635600	-1.13089800
H	-3.42418800	-3.08025600	-2.02279700
N	3.07706700	-0.51940900	-0.26983000
C	4.45912500	-0.66014900	-0.71360000
H	4.93596200	-1.50855100	-0.20083100
H	4.46378500	-0.89504000	-1.77901800

C	5.45820200	0.55764800	-0.49242000
O	5.16047700	1.39963800	0.37961600
O	6.47946500	0.48108700	-1.21671300

(4) TS4

C	-0.70110900	1.13634400	-0.28464800
C	0.61154000	0.38764900	0.03000800
H	-0.60307600	1.67880400	-1.22772900
H	-0.93231200	1.86547500	0.49630200
H	0.50708900	-0.17557000	0.96169400
H	0.83262000	-0.35191200	-0.74612900
C	1.79203200	1.35505200	0.15147100
H	1.90021600	1.93245800	-0.77907200
H	1.57272500	2.10539900	0.92527400
C	3.13767000	0.74434100	0.46168000
C	-1.86466500	0.19076100	-0.40601900
O	-3.14045200	1.55017100	-1.18159000
H	3.93514000	1.47103000	0.67406500
H	-1.86575000	-0.48271000	-1.26049500
C	-3.40120900	-1.38451700	0.67857000
H	-2.83028600	-2.27503700	0.39592100
H	-3.72184000	-1.53750100	1.70978900
H	-2.75393400	0.58995200	1.29465300
H	-3.87015200	0.88051100	-1.25661300
N	-2.49656800	-0.23070700	0.67532000
C	-4.69615900	-1.39891600	-0.21713300
O	-5.45758900	-2.34678300	0.05403100
O	-4.82123600	-0.52452500	-1.11469000
O	-3.23761400	2.22859600	1.26201700
H	-4.08511100	2.48024200	1.63892200
H	-3.27950700	1.97039200	-0.22564000

N	3.36021100	-0.50245200	0.46302100
C	4.71909900	-0.94754500	0.73700500
H	4.72443400	-2.03351700	0.83757200
H	5.07790600	-0.53185500	1.69130200
C	5.84601100	-0.55822200	-0.31755300
O	6.84748700	-1.31119400	-0.26176600
O	5.64528700	0.45326800	-1.02031400

2. Cartesian coordinates for mPA

(1) TS1

C	1.16822700	-0.95213800	-0.50097600
O	1.71170700	-2.20324300	-0.60489100
H	1.35357500	-0.29124400	-1.34962800
C	1.67947000	1.13885300	1.14144100
H	0.72325000	1.12161200	1.67131700
H	2.46043800	1.38015100	1.86414100
H	1.85640000	-0.89904000	1.44483100
H	2.80988500	-2.03290100	-0.61961400
N	1.96331800	-0.25452100	0.65954300
C	1.60456000	2.29360100	0.07853900
O	1.75354200	3.41585300	0.59303400
O	1.34678000	1.96065400	-1.10003700
O	3.99421200	-1.41260800	-0.42710600
H	2.98007900	-0.42843600	0.33648600
H	4.70448500	-1.93059000	-0.04205700
C	-1.13683100	0.10536300	-0.44014100
C	-2.50427600	0.05247300	-0.14615900
C	-3.06091400	-1.10706100	0.41469100

C	-2.24708000	-2.20223200	0.66688700
C	-0.88106800	-2.14815900	0.36921300
C	-0.31363600	-0.99360700	-0.17402500
H	-0.69076500	0.99632100	-0.87717200
H	-4.12262200	-1.12635800	0.63275400
H	-2.67060200	-3.10900700	1.08652400
H	-0.23928800	-3.00677000	0.52774300
C	-3.34522400	1.23027600	-0.44047400
H	-2.79509900	2.08824200	-0.87650900
O	-4.54278400	1.30377700	-0.23969800

(2) TS2

C	-4.06455800	-1.59128900	0.12600300
C	0.68858400	-0.25515300	-0.12472700
O	1.13823100	0.43062100	1.66176800
H	-3.61799600	-2.59612100	0.29017300
H	0.84304800	-1.30470700	0.10968200
C	2.86794800	-0.35889300	-1.21229700
H	2.68816200	-1.18647900	-1.90554900
H	3.48554900	0.36394500	-1.74653700
H	1.63228000	1.36087300	-0.77186900
H	2.02661400	-0.04003100	1.62747500
N	1.58743200	0.30236800	-0.92467300
C	3.74942300	-0.95094800	-0.04839600
O	4.81456900	-1.43507300	-0.45646100
O	3.30247600	-0.91063200	1.13261800
O	1.51199700	2.57999500	0.30662700
H	2.25632700	3.16585000	0.46568200
H	1.33319900	1.38434900	1.30089200
C	-2.51105500	1.80779400	-0.35966200
C	-3.47680200	0.81781200	-0.22537200

C	-3.07724000	-0.51066200	-0.03117500
C	-0.74006800	0.16745400	-0.13167000
C	-1.15107800	1.49591600	-0.31563800
H	-2.81141700	2.84174500	-0.49178800
H	-0.39381800	2.27602400	-0.36855200
H	-4.53554900	1.04765700	-0.25941800
O	-5.27282000	-1.46233000	0.08869300
C	-1.71241800	-0.82155100	0.01924200
H	-1.41047500	-1.85320600	0.17871400

(3) TS3

C	-2.41190600	-0.72159000	0.68327500
O	-3.07544000	-1.83283200	1.13708000
H	-2.34419600	0.09938100	1.40288300
C	-2.97212200	1.21348200	-1.05685500
H	-1.93169800	1.18002700	-1.38825300
H	-3.60484700	1.32540100	-1.93967000
H	-3.30853100	-0.82258900	-1.17960600
H	-4.15493000	-1.60836000	1.13173000
N	-3.31060500	-0.11295100	-0.44555200
C	-3.17343500	2.46750600	-0.13520400
O	-3.18667700	3.53455600	-0.78491900
O	-3.26545700	2.25094300	1.08870400
O	-5.38394600	-1.09318200	0.83491700
H	-4.29766500	-0.17802600	-0.03045300
H	-5.95798700	-1.73535900	0.40821300
C	0.05934100	-0.24654100	0.26915700
C	1.30983600	-0.58044400	-0.27611500
C	1.43125500	-1.77735700	-0.99697200
C	0.33272700	-2.61373400	-1.15728400
C	-0.90326500	-2.27416000	-0.60598600

C	-1.05167700	-1.07822300	0.10335400
H	-0.04725000	0.67519100	0.83445200
H	2.40310300	-2.02989800	-1.40374600
H	0.44257500	-3.54976800	-1.69782500
H	-1.75161900	-2.94555200	-0.67844800
C	2.47248500	0.31347300	-0.09319600
H	2.34170800	1.12641200	0.63662200
N	3.55205700	0.16968800	-0.74935400
C	4.66225500	1.04477500	-0.47689700
H	4.33532800	2.00472800	-0.04128000
H	5.19816600	1.24199400	-1.40596600
C	5.72976900	0.45522600	0.56407100
O	5.25664500	-0.16371700	1.53417600
O	6.91438800	0.75483000	0.28849300

(4) TS4

C	-2.94205900	-0.10321200	0.50905600
C	1.98401200	-0.21474200	-0.04932200
O	2.75867400	0.60263400	1.57814900
H	-2.82479600	-1.16932400	0.75304400
H	1.85192900	-1.21142700	0.36324600
C	3.97789600	-1.12947500	-1.13214900
H	3.48355200	-2.03142600	-1.50889200
H	4.63988500	-0.78643100	-1.92819800
H	3.31449000	0.90956600	-0.99217100
H	3.49627900	-0.06888900	1.61143400
N	2.96197600	-0.08781600	-0.94352200
C	4.89422300	-1.60984800	0.05492500
O	5.82836200	-2.33975000	-0.32409200
O	4.59147100	-1.27292100	1.23143600
O	3.68234000	2.30178900	-0.08438500

H	4.61343600	2.52266100	0.00670000
H	3.18106800	1.39800600	1.03587500
N	-4.07069300	0.47820600	0.58593900
C	-5.22856600	-0.30146300	0.94507300
H	-5.98202000	0.35274100	1.38405800
H	-4.97892900	-1.08943800	1.67570200
C	-5.92540300	-1.08917900	-0.26484200
O	-5.15436100	-1.58339400	-1.10717300
O	-7.17145600	-1.15916900	-0.16035900
C	-0.52491500	2.57422500	-0.64325500
C	-1.71602700	1.93033800	-0.32075900
C	-1.70259300	0.60051500	0.12118200
C	-0.46628400	-0.05300200	0.21450700
C	0.73359800	0.58586100	-0.11953600
C	0.70206300	1.91880200	-0.54819700
H	-0.54680900	3.60950600	-0.97087200
H	-0.44441500	-1.08498700	0.55461000
H	1.63381200	2.43586800	-0.75472700
H	-2.67237900	2.43441600	-0.39544300

3. Cartesian coordinates for DFF

(1) TS1

C	-0.33510700	-0.90697700	0.37247700
H	0.44115800	-1.65973400	0.20655800
C	-2.54017900	-0.95694800	-0.47671900
H	-3.01085400	-1.70110000	-1.13133100
C	-3.27206600	0.37382600	-0.65817300
C	0.35355800	0.43450600	0.65579600
O	-0.54008000	1.45363400	0.86057300

H	-2.64300700	1.25756500	-0.44307400
H	1.09302800	0.29014100	1.44574300
C	2.27405500	-0.03566200	-1.15622000
H	1.79199700	-0.80235200	-1.76776400
H	2.85975500	0.60118400	-1.82029400
H	0.47552700	0.98027700	-1.30423500
H	0.07672300	2.37546100	0.80049200
N	1.18539400	0.83522000	-0.58365500
C	3.25944800	-0.77692100	-0.17733200
O	4.26349800	-1.20395200	-0.77629000
O	2.90532600	-0.89335700	1.01500600
O	1.11884100	3.18401200	0.49420000
H	1.48983800	1.83316000	-0.31577900
H	0.90657900	4.04261900	0.12147300
O	-1.16117500	-0.82325600	-0.81855000
C	-1.29542900	-1.31552900	1.44927100
C	-2.53703200	-1.35998100	0.97876800
H	-0.97736600	-1.52361300	2.46190500
H	-3.44018500	-1.60012000	1.52328200
O	-4.43696400	0.44818000	-0.97726300

(2) TS2

C	0.90202400	0.94575000	0.04264300
H	0.62490600	1.96009800	-0.26097800
C	3.10118700	0.12220700	-0.20002600
H	3.92125700	0.71203800	-0.63453400
C	3.46352700	-1.35366600	-0.34109300
C	-0.35985300	0.09792300	0.07267200
O	4.58935300	-1.78102300	-0.26043900
O	-0.94658500	0.54448100	-1.73728500
H	2.58941800	-2.01777600	-0.50548600

H	-0.21235300	-0.96585000	-0.09986700
C	-2.45559400	-0.40172900	1.20923100
H	-2.08463100	-1.18056900	1.88365200
H	-3.17236900	0.18756700	1.78304700
H	-1.58199800	1.54960800	0.60460300
H	-1.75483600	-0.05522500	-1.64128300
N	-1.34756500	0.49273700	0.85342400
C	-3.26814700	-1.14951100	0.08836500
O	-4.21800200	-1.80694300	0.53786000
O	-2.88323300	-1.04585400	-1.11171400
O	-1.55320400	2.69041100	-0.36675600
H	-2.33721200	3.23594900	-0.46689100
H	-1.26898300	1.46295600	-1.44288000
O	1.87938400	0.39402800	-0.86861000
C	1.60849300	0.93653500	1.37458900
C	2.84878900	0.47772500	1.24747000
H	1.12794500	1.28301500	2.27876600
H	3.59756200	0.36683000	2.02032400

(3) TS3

C	1.06550100	-0.76474200	0.85627100
H	1.72903600	-1.63830300	0.75165400
C	-1.19832300	-1.14338100	0.25029700
H	-1.44148800	-2.15236200	-0.11019400
C	-2.26431500	-0.21569000	-0.27828000
C	1.94906300	0.49513800	0.81245100
O	1.28338300	1.63630700	1.13910600
H	-2.14024400	0.84891500	-0.03444500
H	2.86999000	0.31388800	1.37792300
C	3.29969700	-0.37196000	-1.31669800
H	2.69945900	-1.27596100	-1.43196200

H	3.51924200	0.00443800	-2.31832600
H	1.56799100	0.74748200	-1.17521100
H	1.88691100	2.49227900	0.71317300
N	2.44288000	0.66538200	-0.65085600
C	4.65975600	-0.75765900	-0.63531000
O	5.33532500	-1.52485500	-1.35612900
O	4.89265600	-0.29340500	0.49816400
O	2.70674400	3.22590400	-0.02251600
H	2.85260900	1.64737500	-0.64148700
H	2.21409600	3.89463900	-0.50630500
N	-3.25133700	-0.67575300	-0.92458500
C	-4.32346600	0.21974500	-1.29605700
H	-3.98816000	1.27000800	-1.34096300
H	-4.70551600	-0.06928500	-2.27604100
C	-5.55936400	0.21065400	-0.28634600
O	-5.28731600	0.04475000	0.91822400
O	-6.65993500	0.43352300	-0.84383700
O	0.11849700	-0.77185000	-0.22865300
C	0.21488400	-0.88878000	2.08936900
C	-1.04997900	-1.11212500	1.75161600
H	0.61748100	-0.80104600	3.08988100
H	-1.89775600	-1.23966700	2.41163500

(4) TS4

C	-0.73023200	1.20505900	0.13520000
H	-1.23625900	2.15687600	-0.06547200
C	1.61329500	1.20151600	-0.15823900
H	2.03357600	2.16066200	-0.49347700
C	2.61265600	0.12864800	-0.51406300
C	-1.75882400	0.09000500	0.04766100
O	-2.58486200	0.68810500	-1.65836200

H	2.28501700	-0.90805700	-0.34667800
H	-1.37267500	-0.86178400	-0.30844800
C	-3.61658000	-1.09170400	1.10699100
H	-2.97487300	-1.92412300	1.41570200
H	-4.27099200	-0.87211100	1.95179300
H	-3.26694700	1.05674600	0.86169200
H	-3.25655700	-0.05292100	-1.64862300
N	-2.77763700	0.08971100	0.88843900
C	-4.52475300	-1.65173000	-0.04971300
O	-5.38012800	-2.46233300	0.35155300
O	-4.28524500	-1.28790100	-1.23294400
O	-3.62522200	2.36882800	0.03486900
H	-4.55448500	2.60359500	-0.03643000
H	-3.07101200	1.45161900	-1.17349400
O	0.33726000	0.97649000	-0.79615000
C	-0.03757900	1.23861500	1.48136300
C	1.27972700	1.24503600	1.31319800
N	3.77130100	0.43278800	-0.92238600
C	4.74825200	-0.61965100	-1.08586900
H	5.43526500	-0.35240100	-1.88929200
H	4.27535900	-1.58576000	-1.33085400
C	5.62257300	-0.90170900	0.22004500
O	5.03303900	-0.78267300	1.31176800
O	6.79361700	-1.27001300	-0.03127900
H	2.04764700	1.28042200	2.07468700
H	-0.58759600	1.28121900	2.41143800

4. Cartesian coordinates for PDA

(1) TS1

C	0.96782800	0.90360400	0.66182500
O	0.89854100	2.25516900	0.86492500
H	1.64377600	0.36469100	1.32486900
C	1.80366600	-0.67050100	-1.33388500
H	0.85109300	-1.01179700	-1.74602100
H	2.50209200	-0.54137400	-2.16115700
H	0.92572700	1.21077200	-1.35350900
H	1.91852900	2.62353700	0.56756100
N	1.57249800	0.69885700	-0.74938200
C	2.33328700	-1.82757900	-0.40524100
O	2.80451400	-2.76935000	-1.06762900
O	2.16664400	-1.69936900	0.82740600
O	3.11840500	2.65164300	-0.01199400
H	2.44230600	1.32391800	-0.69380600
H	3.34036700	3.47685300	-0.44942200
C	-2.51048500	0.10182500	-0.21414700
C	-2.95784900	-0.74284100	0.80307300
C	-2.05239700	-1.10633000	1.79432100
C	-0.75824100	-0.60101700	1.74051200
C	-0.40994000	0.27083200	0.70222100
H	-3.97919300	-1.10283300	0.78564200
H	-2.34468300	-1.78438500	2.58862400
H	-0.00211200	-0.88973300	2.45881400
C	-3.40248700	0.51333900	-1.32820500
H	-2.90148100	1.16546700	-2.07049600
N	-1.26486600	0.59757500	-0.26837100
O	-4.56687100	0.19214000	-1.44391800

(2) TS2

C	-3.92001800	-1.64322200	0.11491400

C	0.62791500	-0.28889800	-0.08833600
O	1.12179100	0.53203800	1.63226200
H	-3.39838200	-2.61118800	0.24813200
H	0.72593000	-1.32576600	0.21529600
C	2.77999500	-0.52447500	-1.20058200
H	2.55672900	-1.45240200	-1.73590500
H	3.36386500	0.09686800	-1.87982200
H	1.58625200	1.25213600	-0.88558900
H	2.01027700	0.05220300	1.61278800
N	1.51974200	0.18503700	-0.94369700
C	3.72503100	-0.92962700	-0.00634200
O	4.80391000	-1.40117900	-0.39103100
O	3.30409900	-0.78434400	1.17600100
O	1.50725800	2.57097300	0.07686900
H	2.26549300	3.15347300	0.16753400
H	1.32558200	1.44504100	1.20222800
C	-2.52237000	1.83151700	-0.29523900
C	-3.46205200	0.81051600	-0.18036900
C	-2.99213600	-0.49235700	-0.02324600
C	-0.79355300	0.17879100	-0.09963900
C	-1.16813200	1.52470600	-0.25743200
H	-2.83987400	2.86290000	-0.40583700
H	-0.39012400	2.28768500	-0.30510800
H	-4.52961500	0.99323000	-0.20202700
N	-1.68705800	-0.80629100	0.01888900
O	-5.13118500	-1.56837900	0.08834900

(3) TS3

C	-2.27018500	0.84614900	0.43824200
O	-2.40007200	1.52019000	1.62763300
H	-3.07652000	1.06820700	-0.27481700

C	-2.79946200	-1.49881300	-0.45643500
H	-2.14660600	-1.27869300	-1.30401800
H	-2.62519700	-2.53942900	-0.17349800
H	-1.48173100	-0.93949800	1.04636100
H	-3.17605200	0.93034900	2.22003300
N	-2.40098100	-0.64921900	0.70291700
C	-4.29711300	-1.36456300	-0.90482400
O	-4.56955900	-2.08243400	-1.89103000
O	-5.02032300	-0.58911400	-0.24261400
O	-3.89601400	-0.06229400	2.68827800
H	-3.12930300	-0.66233600	1.55313200
H	-4.75656300	-0.03518300	2.25269500
C	1.32191500	0.59003200	-0.42407700
C	1.55960300	1.71868400	-1.22080500
C	0.50037700	2.56798500	-1.50534700
C	-0.76213300	2.27711700	-0.99332400
C	-0.91322700	1.14297700	-0.19501900
H	2.56233200	1.88655200	-1.59151700
H	0.65361600	3.44955400	-2.12084800
H	-1.61735100	2.91325800	-1.18991000
C	2.40293500	-0.37288600	-0.12199000
H	2.17819200	-1.09237800	0.67640200
N	0.10424100	0.32111800	0.08333200
N	3.49486300	-0.37562300	-0.77318300
C	4.52873300	-1.30733900	-0.41287500
H	5.03518300	-1.64580900	-1.31723900
H	4.13039200	-2.18362900	0.12618700
C	5.66236000	-0.70174500	0.55263000
O	5.27028400	0.10379000	1.41400400
O	6.79817400	-1.18144800	0.33729400

(4) TS4

C	-2.80132000	-0.25793000	0.48795700
C	1.93778300	-0.22365400	-0.01608000
O	2.77235700	0.65712800	1.55160500
H	-2.62008700	-1.33529900	0.59688700
H	1.76734700	-1.19570600	0.43489300
C	3.88236700	-1.20531900	-1.12106600
H	3.36008800	-2.12855900	-1.39260600
H	4.49301500	-0.92806200	-1.98098900
H	3.24209800	0.84707000	-1.05428800
H	3.50831700	-0.01939300	1.58276300
N	2.88447100	-0.14455600	-0.94323000
C	4.86877500	-1.59679400	0.04251400
O	5.79996300	-2.32762500	-0.34089900
O	4.61492600	-1.19978000	1.21183200
O	3.64867100	2.28241000	-0.23614400
H	4.58032100	2.51501200	-0.19532500
H	3.18125100	1.41948100	0.97057400
N	-3.93928100	0.27714400	0.67480800
C	-5.06846200	-0.56337400	0.97460900
H	-5.73172500	-0.03852500	1.66290400
H	-4.76255000	-1.51992000	1.43178600
C	-5.95285800	-0.97782700	-0.29883800
O	-5.32517300	-1.20833200	-1.34725300
O	-7.17452700	-1.07538300	-0.04099700
C	-0.54928100	2.59397500	-0.51527700
C	-1.71072400	1.89055200	-0.22637700
C	-1.60642300	0.54224500	0.13990700
C	0.69405400	0.60567900	-0.07733500
C	0.68567600	1.95427900	-0.44520300

H	-0.59887200	3.64219600	-0.79489900
H	1.62370200	2.47029700	-0.62900100
H	-2.69493600	2.33950200	-0.26840500
N	-0.41879200	-0.08493500	0.20866000
